# Psychometric Properties of Instruments to Assess Movement Disorders in People With Intellectual Disabilities Using Antipsychotics: A Systematic Literature Review

**DOI:** 10.1111/jar.70303

**Published:** 2026-09-08

**Authors:** Maria Louise Dieudonnée Hoekstra‐van Duijn, Maureen Bernardina Gerhardus Wissing, Bibi den Haan, Sylvie Beumer, Dederieke Anne Maria Maes‐Festen

**Affiliations:** ^1^ Department of General Practice, Intellectual Disability Medicine Research Erasmus MC, University Medical Center Rotterdam the Netherlands; ^2^ Academic Collaborative Research Center Healthy Ageing and Intellectual Disabilities Rotterdam the Netherlands

**Keywords:** antipsychotics, instrument, intellectual disabilities, movement disorders, psychometry

## Abstract

**Background:**

Assessing movement disorders—side effects of antipsychotics—can be challenging in people with intellectual disabilities. This systematic literature review aims to provide a comprehensive overview of the psychometric properties of instruments used to assess movement disorders in people with intellectual disabilities using antipsychotics.

**Methods:**

A comprehensive search was conducted across multiple databases. Results of psychometric properties for each instrument in the included studies were extracted.

**Results:**

Ten studies were included, evaluating psychometric properties of seven instruments (DISCUS, AIMS, CLAMPS, ARMS, checklist for motor restlessness, MEDS and analogue functional analyses). There was not a singular instrument that consistently demonstrated good feasibility, interrater reliability and validity across studies.

**Conclusions:**

Findings suggest that instruments lack evidence of good psychometric quality for assessing movement disorders in people with intellectual disabilities using antipsychotics. Future research should focus on the evaluation of psychometric properties of existing instruments and develop and validate novel instruments.

## Summary

- Antipsychotics are commonly used by people with intellectual disabilities and can cause movement disorders that can lead to discomfort and a lower quality of life.
- Early identification is important, but can be challenging because people with intellectual disabilities may have difficulty describing their symptoms, and the movement disorders may be mistaken for challenging behaviour or other health conditions.
- This review examines the available evidence on instruments used to assess movement disorders in people with intellectual disabilities who use antipsychotics.

## 1 Introduction

The use of antipsychotics is prevalent among people with intellectual disabilities, a group defined by limitations in both intellectual functioning and adaptive behaviour (Schalock et al. 2021). In a recent systematic review a pooled prevalence of 31% for the use of antipsychotics among people with intellectual disabilities was reported (Song et al. 2023). Reasons for antipsychotic prescription may be diverse and can include the treatment of psychotic disorders and challenging behaviour, despite the lack of evidence supporting their efficacy in challenging behaviour (Brylewski and Duggan 2004; Charlot et al. 2020). Using antipsychotics may lead to side effects that could negatively impact the quality of life of people with intellectual disabilities (Scheifes et al. 2016a).

One of those side effects is the onset of movement disorders, also referred to as extrapyramidal symptoms. Movement disorders arise due to a blockage of dopamine receptors in the basal ganglia. These brain structures play an important role in the regulation of movement (Rissardo et al. 2023). Movement disorders can develop acutely (within minutes to days), subacutely (within days to weeks) or tardively (after weeks or even years) following the initiation or dose adjustment of antipsychotics (Duma and Fung 2019). Previous studies have indicated that approximately 44%–53% of people with intellectual disabilities using antipsychotics had at least one movement disorder (de Kuijper et al. 2013; Scheifes et al. 2016b).

Four potential antipsychotic‐induced movement disorders are akathisia, dyskinesia, dystonia and parkinsonism. Akathisia is a sense of internal restlessness and irritability, often accompanied by physical signs and the inability to sit still (Duma and Fung 2019; Rissardo et al. 2023). Dyskinesia presents as involuntary, abnormal and repetitive movements that can affect various body parts, including the facial muscles (Rissardo et al. 2023). Dystonia involves uncontrolled, involuntary and sometimes painful muscle spasms that can affect any muscle group, such as the neck, face or limbs (Duma and Fung 2019; Rissardo et al. 2023). Parkinsonism encompasses a range of clinical features including bradykinesia (slowness of movement), rigidity and resting tremor (Duma and Fung 2019; Rissardo et al. 2023). These movement disorders can be distressing and interfere with daily functioning (Duma and Fung 2019; Rissardo et al. 2023).

As movement disorders are associated with a reduced quality of life (Adrianzén et al. 2010; Scheifes et al. 2016a) and clinicians may change treatment plans because of this (Scheifes et al. 2016a), early recognition is essential. This would allow for timely intervention, that is, dose reduction, discontinuation or switching to an antipsychotic with a lower risk of causing these side‐effects (Duma and Fung 2019). Furthermore, early detection of movement disorders and subsequent modification of treatment may reduce the likelihood of progression to persistent movement disorders (Caligiuri and Lohr 1990).

However, adequately detecting antipsychotic‐induced movement disorders can be challenging in people with intellectual disabilities, particularly when there may be limited capacity to understand, express or communicate symptoms to caregivers or clinicians. Furthermore, symptoms of movement disorders may be mistaken for other physical or mental health comorbidities due to overlap in clinical presentation or even attributed to behaviours that challenge (Kelly et al. 2022; Sheehan et al. 2017). Using standardised instruments to assess antipsychotic‐induced movement disorders can help clinicians identify and monitor these side effects in people with intellectual disabilities.

Several instruments have been developed to assess antipsychotic‐induced movement disorders in the general population, for example the St. Hans Rating Scale for akathisia, dyskinesia, dystonia and parkinsonism (Gerlach et al. 1993), the Abnormal Involuntary Movement Scale (AIMS) for dyskinesia (Guy et al. 1976) and the Barnes Akathisia Rating Scale for akathisia (Barnes 1989). Insight into the psychometric properties of instruments is crucial to ensure the quality of instruments (Ohiri and Nnennaya 2024) and provides evidence‐based guidance for the selection of instruments with good psychometric properties for clinical practice. The aforementioned instruments have been researched for their psychometric properties in the general population and had sufficient reliability and validity (Barnes 2003; Gerlach et al. 1993; Lane et al. 1985). However, psychometric properties of instruments are population‐specific and cannot automatically be transferred to people with intellectual disabilities.

Research on instruments for measuring side effects across all types of medication (not solely antipsychotics) in people with intellectual disabilities and the available evidence on the psychometric properties was summarised in a scoping review by (Kelly et al. 2022). They only considered rating scales—as opposed to all types of instruments—and solely included studies published in the previous 20 years. There is, to the best of our knowledge, no comprehensive overview of the psychometric properties of assessment antipsychotic‐induced movement disorders in people with intellectual disabilities.

Considering the importance of early detection of movement disorders in people with intellectual disabilities, this systematic literature review therefore aims to provide a comprehensive overview of the psychometric properties of instruments used to assess movement disorders in people with intellectual disabilities using antipsychotics.

## 2 Methods

This systematic literature review was registered in PROSPERO (CRD42020152399). Findings are reported in accordance with the Preferred Reporting Items for Systematic Reviews and Meta‐Analyses (PRISMA) guidelines (Page et al. 2021).

### 2.1 Search Strategy

A comprehensive literature search was conducted across five databases: Embase, MEDLINE, Web of Science, Cochrane Central Register of Controlled Trials and PsycINFO Ovid. The search employed a combination of search terms and Boolean operators to create a comprehensive search strategy: ‘intellectual disability’ AND (‘dystonia’ OR ‘akathisia’ OR ‘parkinsonism’ OR ‘dyskinesia’ OR ‘extrapyramidal symptoms’) AND (‘drug‐induced’ OR ‘antipsychotic’) AND ‘psychometry’ along with medical subject headings (MeSH terms) when available, synonyms, alternative spellings and commonly used technical terms for psychometric properties. Asterisks (*) were used to truncate search terms to account for variations in spelling. Duplicates were removed using Endnote (The EndNote Team 2013). The full search strategy is provided in Supporting Information A. The search was developed and executed by an information specialist with expertise in systematic review searching from the Medical Library of Erasmus MC, University Medical Center, Rotterdam, the Netherlands. The search spanned from the inception of each database through October 8, 2019 and was updated on March 10, 2025.

Additionally, trial registries (ClinicalTrials.gov, International Clinical Trials Registry Platform, EU clinical Trials Register, ISRCTN registry and International Traditional Medicine Clinical Trial Registry) were searched on April 23, 2025 for ongoing or completed trials related to antipsychotic‐induced movement disorders in people with intellectual disabilities using the following keywords: (‘movement disorder’ OR ‘dyskinesia’ OR ‘dystonia’ OR ‘akathisia’ OR ‘acathisia’ OR ‘parkinsonism’ OR ‘bradykinesia’) AND ‘intellectual disability’ (no date or language limit applied). The retrieved trials were reviewed by one reviewer (MHvD) for relevant records.

### 2.2 Study Selection

Studies were included if they reported psychometric properties of instruments used to assess movement disorders in people with intellectual disabilities using antipsychotics and were published in English. Eligible studies involved participants with intellectual disabilities (IQ < 70) who were using antipsychotics, encompassing both children and (older) adults. Studies had to report on psychometric properties (feasibility, reliability, validity, internal consistency, measurement error, hypothesis testing for construct validity, responsiveness) of instruments used to assess antipsychotic‐induced movement disorders, regardless of whether this was their primary objective. There was no restriction on the type of instrument, for example questionnaires, observational checklists, (electronic) devices, scales, sensors or other diagnostic instruments. Studies were only included if results on psychometric properties were reported separately for participants with intellectual disabilities using antipsychotics or if a group with intellectual disabilities not using antipsychotics was employed to examine validity. Studies that did not explicitly report the use of antipsychotics but did provide chlorpromazine (CPZ) equivalents were included based on the rationale that CPZ equivalents are calculated for antipsychotics (Rijcken et al. 2003). In contrast, studies referring to ‘neuroleptic use’ without providing CPZ equivalents or without clarifying the specific medications involved were excluded.

Reasons for exclusion were (in this order): full‐text report not available; report not in English; no results reported (‘non‐original research’, e.g., congress abstract, systematic or scoping reviews); study population did not only comprise people with intellectual disabilities (IQ < 70) (or results were not reported separately); no report on movement disorders; no assessment of movement disorders; no antipsychotic use (or results were not reported separately for participants using antipsychotics); and no report on psychometric properties of the instrument used to assess movement disorders.

### 2.3 Selection Process

Study selection was conducted in two stages. First, reviewing titles and abstracts to identify potentially eligible records was independently executed by two pairs of reviewers (original search MHvD and PH; updated search MHvD and MW) using Rayyan (Ouzzani et al. 2016). Hereafter, the reviewers held a consensus meeting to determine which records should proceed to full‐text screening. In the second stage, two reviewers (MHvD and BdH) independently read all full‐text reports and assessed the eligibility based on predefined inclusion and exclusion criteria. Reasons for exclusion were tracked in EndNote (The EndNote Team 2013) following the method described previously (Bramer et al. 2017). Disagreements were resolved by consensus discussion and if necessary, a third reviewer was consulted. The reasons for exclusion were documented.

To further identify eligible records, the reference lists of relevant (systematic) reviews on antipsychotic or psychotropic use in people with intellectual disabilities were reviewed by one reviewer (MHvD), along with the reference lists of studies included in this review. The references with (a synonym of) intellectual disabilities, antipsychotics and side effects/movement disorders in the title were eligible for screening of title and abstract. This was executed by two reviewers (MHvD and MW).

### 2.4 Data Collection

The primary outcomes of this review were the psychometric properties of instruments that were used to assess movement disorders in people with intellectual disabilities using antipsychotics. A standardised data extraction form (Supporting Information B) developed for this systematic literature review was used for data extraction. One reviewer (MHvD) extracted study (author, year of publication, study design, primary objective(s), sample size), participant (age, sex, level of intellectual disabilities, aetiology of intellectual disabilities, antipsychotic use) and instrument characteristics (name of instrument, description of instrument, type of psychometric property, description of psychometric property, method of analysis of psychometric property and results of analysis of psychometric property). When multiple psychometric properties were reported, only the psychometric properties that were examined in people with intellectual disabilities using antipsychotics were extracted. A second reviewer (BdH) verified the extracted data. Disagreements were resolved through consensus discussions.

### 2.5 Quality Assessment

We assessed the quality of the examinations of psychometric properties reported in the included studies with the Standard Quality Assessment Criteria for Evaluating Primary Research Papers from a Variety of Fields (Kmet et al. 2004). Fourteen items were scored ‘yes’ (2), ‘partial’ (1), ‘no’ (0) or ‘not applicable’. The sum of the scores on the applicable items was divided by the maximum possible score. This resulted in the summary score ranges from 0–1.0, a higher score represents higher quality. When multiple instruments were examined within one study we evaluated per psychometric property rather than per instrument, that is, when feasibility of two instruments was examined within one study, we evaluated the examination of feasibility once. One reviewer (MHvD) extracted the data, which was verified by a second reviewer (BdH). Disagreements were resolved through consensus discussions. The extracted data was summarised both in tables and in narrative form.

### 2.6 Data Synthesis

Data synthesis of the results of the psychometric properties was performed for each included study. The psychometric properties, as mentioned in the original report, were converted into uniform terminology: feasibility, interrater reliability and validity (Mokkink et al. 2010). Results of the psychometric properties are summarised in both narrative and tabular form, using the guidelines for interpretation presented in Table 1 to facilitate comparison across studies. The results are presented per movement disorder and then according to the frequency of examination of the instrument.

**TABLE 1:** Guidelines for interpretation of results of psychometric properties.

	Interpretation	
Quartiles (feasibility) (Hilgenkamp et al. 2013)		
< 25%	Low	−
25%–50%	Moderate	+/−
50%–75%	Good	+
> 75%	Excellent	++
Correlations (interrater reliability)^a^ (Terwee et al. 2007)		
< 0.70	Negative rating	−
> 0.70	Positive rating	+
Spearman's correlation coefficient (validity) (Hazra and Gogtay 2016)		
< 0.30	Poor correlation	−
0.30–0.50	Fair/Moderate correlation	+/−
0.50–0.70	Good correlation	+
> 0.7	Strong correlation	++

^a^ If mean reliability score was not reported, range was utilised.

## 3 Results

### 3.1 Selection of Studies

**FIGURE 1:**
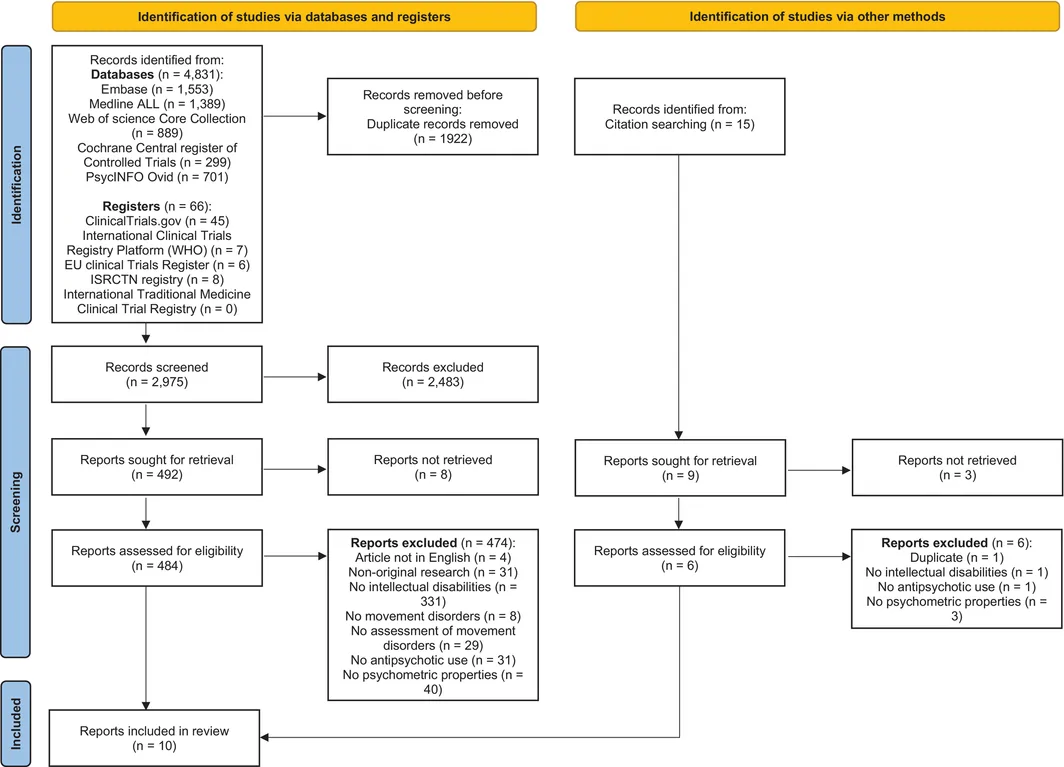
Flowchart of study selection process.

Figure 1 presents a flowchart of the study selection process. A total of 4831 records were retrieved from five databases. The search of trial registries resulted in 66 records. After removing duplicates, 2975 records were screened on title and abstract, which resulted in 492 reports. After full‐text screening, a total of 10 studies were included.

Screening of the reference lists of the included studies, relevant systematic reviews and other relevant reports revealed 15 additional possibly eligible reports. Upon enquiry with the Medical Library, it was clarified that these additional reports did not contain any of the psychometric‐related search terms and therefore were not retrieved through the electronic search of the five databases. None of those 15 additional reports met the inclusion criteria; six were excluded based on title/abstract screening, three reports could not be retrieved and six were excluded after full‐text screening. As a result, a total of 10 studies were included in this systematic literature review.

### 3.2 Description of the Included Studies

Table 2 presents details of the 10 included studies. The year of publication ranged from 1989 to 2016. The primary objectives were diverse; for example, examining the effects of drug treatment on movement disorders or examining the prevalence of movement disorders. Three of the included studies had the primary objective to examine psychometric properties of instruments to assess movement disorders in people with intellectual disabilities using antipsychotics (Bodfish et al. 1997; Ellis et al. 1996; Garcia and Matson 2008). The number of participants ranged from 2 to 94 and age ranged from 25 to 83 years. Participants had levels of intellectual disabilities ranging from mild to profound. The aetiology of intellectual disabilities was reported in two studies. The studies of Ricketts et al. (1995) and Ellis et al. (1996) were performed within the same study population. There were studies that examined one psychometric property of one instrument or one psychometric property of multiple instruments and there were studies that examined multiple psychometric properties of one or multiple instruments. In some studies, the entire study population was included for the examination of psychometric properties, whereas in other studies the examination was performed in a subset of the total study population.

**TABLE 2:** Details of included studies and main results of examination of psychometric properties.

References	Primary study objective(s)	Participants	Instrument	Psychometric properties
		Number of participants: *n*; mean age (SD); range age; sex; AP use; level of ID; aetiology of ID		
Advokat et al. (2000)	Comparison of side effects	*n* = 51; mean age NR; range age NR; 31 ♀; 34/51 AP; moderate 8, severe 9, profound 31, unspecified 3; aetiology NR	MEDS subscales CNS‐BA; CNS‐D; and CNS‐PD	Specificity (indicated by differences in ratings among subscales): Significant and clinically relevant differences between groups taking atypical, typical and no AP medication for CNS‐BA (*p* < 0.009). No significant differences between groups taking atypical, typical and no AP medication for CNS‐D (*p* = 0.317) and CNS‐PD (*p* = 0.250).
Bodfish et al. (1997)	Construction of akathisia rating scale; psychometry; phenomenology akathisia	*n* = 20; mean age NR; range age NR; sex NR; 20/20 AP; level of ID NR; aetiology NR	ARMS	Interrater reliability (*n* = 20): Rating presence or absence of movement regardless of severity: mean 86.10% (range 67%–100%). Pearson product–moment ARMS total score 0.69, *p* < 0.05.
Bodfish et al. (1997)	Construction of akathisia rating scale; psychometry; phenomenology akathisia	*n* = 66; 37.1 years. (SD 10); range age NR; 19.6% ♀; 66/66 AP; level of ID NR; aetiology NR		Assessability (*n* = 66): Items 1 to 3 (sitting): 100%. Item 4 (sitting): 96.9%. Items 5 and 6 (standing): 100%. Item 7 (standing): 93.9%. Items 8 to 10 (lying): 62%.
Bodfish et al. (1997)	Construction of akathisia rating scale; psychometry; phenomenology akathisia	*n* = 94; mean age NR; range age NR; sex NR; 74/94 AP; level of ID NR; aetiology NR		Predictive validity (*n* = 94): ARMS validly measured differences: mean score neuroleptic group higher than neuroleptic‐free group (*p* < 0.025). Significant increase in mean ARMS score from baseline to post‐reduction in neuroleptic‐reduction group (*p* < 0.05).
De Kuijper and Hoekstra (2016)	Examination potential usefulness of MEDS	*n* = 71; 50.2 years (SD 14.2); range age NR; 30% ♀; 71/71 AP; mild 17%, moderate 35%, severe 37%, profound 10%; aetiology NR	AIMS DISCUS MEDS subscales CNS‐BA and CNS‐PD	Associations at item and scale level: Significant associations between MEDS subscale ratings and DISCUS and UPDRS ratings of ID physician for 5/22 items. No significant associations between MEDS subscale ratings and AIMS ratings of ID physician. No significant associations between MEDS CNS‐PD subscale and total scores of AIMS + UPDRS and DISCUS + UPDRS. MEDS CNS‐BA subscale scores were at trend level associated with the BARS scale scores (*p* = 0.07). Ratings based on assessments of ID physicians and ratings based on observations of caregivers were more in accordance with each other in the DISCUS group than in the AIMS group.
Ellis et al. (1996)	Psychometry of three rating scales	*n* = 7^a^; mean age NR; range 54–83 years; 5 ♀; 7/7 AP; profound 6, severe 1; Brain damage of unknown aetiology 3, from head injury 1, from Down's mosaicism 1, from Down's syndrome 1, due to pertussis 1	AIMS CLAMPS DISCUS	Interrater reliability AIMS ICC 0.52–0.71. CLAMPS Frequency: ICC 0.41–0.89. Severity: ICC 0.22–0.71. DISCUS ICC 0.40–0.73. Concurrent validity (SCCs with *p* ≤ 0.001) Total AIMS and Total severity CLAMPS: 0.52. Total frequency CLAMPS: 0.53. Total DISCUS: 0.56. CLAMPS total Severity and total frequency: 0.96. Severity and total AIMS: 0.52. Severity and total DISCUS: 0.69. Frequency and total AIMS: 0.53. Frequency and total DISCUS: 0.67. Total DISCUS and Total CLAMPS frequency: 0.67. Total CLAMPS severity: 0.69. Total AIMS: 0.56.
Ganesh et al. (1989)	Prevalence of akathisia	*n* = 5; 36.8 years (SD NR); range 25–45 years; 2 ♀; 5/5 AP; level of ID NR; aetiology NR	Checklist for motor restlessness	Interrater reliability *r* = 0.86 (sitting). *r* = 0.95 (standing). *r* = 0.98 (lying).
Garcia and Matson (2008)	Psychometry of two rating scales	*n* = 66; 52 years (SD NR); range 29–79 years; 54.5% ♀; 66/66 AP; moderate 5, severe 10, profound 51; aetiology NR	ARMS MEDS subscale CNS‐BA	Concurrent validity (ARMS and MEDS): Significant differences between control and akathisia group, and between no akathisia and akathisia group on both measures. No significant differences between control and no akathisia group on both measures. Pearson correlation of 0.51 between MEDS CNS‐BA and ARMS, indicating that they are moderately associated. Concurrent validity (ARMS): Two items that differentiated those with a diagnosis of akathisia from those without. Concurrent validity (MEDS CNS‐BA): Four items that differentiated those with a diagnosis of akathisia from those without.
Matson et al. (2002)	Incidence of tardive dyskinesia	*n* = 25; 41 years (SD NR); range age NR; 15 ♀; 25/25 AP; moderate 3, severe 5, profound 17; aetiology NR	DISCUS	Assessability: Successful administration in 77%–100%.
Newell et al. (2001)	Examination of dyskinesia over time	*n* = 26; 34.9 years (SD NR); range age NR; 8 ♀; 26/26 AP; mild 1, moderate 5, severe 12, profound 8; aetiology NR	DISCUS	Interrater reliability: Interrater correlation 0.84.^b^ Interitem correlations (within and between three testing periods): Significant correlation coefficients within (0.394–0.641) and between (0.396–0.999) testing periods. At baseline and follow‐up, the majority involved grimace, tic, and blinking facial items; at peak withdrawal, significant interitem correlations oro‐bucco‐lingual items. Unique contribution: Significant predictor items were found. Number of items uniquely contributing to total DISCUS score at peak withdrawal is almost twice the number than at baseline of follow‐up.
Ricketts et al. (1995)	Effects of medication on tardive dyskinesia	*n* = 7^a^; mean age NR; range 54–83 years; 5 ♀; 7/7 AP; profound 6, severe 1; Brain damage of unknown aetiology 3, from head injury 1, from Down's mosaicism 1, from Down's syndrome 1, due to pertussis 1	AIMS DISCUS	Interrater reliability (AIMS): Mean 76.4% (range 57.1%–85.7%) Interrater reliability (DISCUS): Mean 80.6% (range 66.6%–93.3%)
Valdovinos et al. (2004)	Examination of dyskinesia in different conditions	*n* = 2; mean age NR; range 52–53 years; 0 ♀; 2/2 AP; mild 2; aetiology NR	Analogue functional analyses	Interobserver agreement: Participant 1: 91% (range 84% to 99%) Participant 2: 98% (range 87% to 100%)

Abbreviations: AIMS, Abnormal Involuntary Movement Scale; AP, antipsychotic; ARMS, Akathisia Ratings of Movement Scale; BARS, Barnes Akathisia Rating Scale; CLAMPS, CLAMPS Abnormal Movement Scale; CNS‐BA, Central Nervous System‐Behavioural/Akathisia; CNS‐D, Central Nervous System‐Dystonia; CNS‐PD, Central Nervous System‐Parkinsonism/Dyskinesia; DISCUS, Dyskinesia Identification System Condensed User Scale; ICC, intraclass correlation coefficient; ID, intellectual disability; MEDS, Matson Evaluation of Drug Side Effects; *n*, number of participants; NR, not reported; SCC, Spearman correlation coefficient; SD, standard deviation; UPDRS, Unified Parkinson's Disease Rating Scale.

^a^ Ricketts et al. (1995) and Ellis et al. (1996) reported on the same study population.

^b^ Analysis of reliability was based on 94 sessions, it is not clear whether all 26 participants were represented in those 94 sessions.

### 3.3 Quality Assessment

The psychometric properties reported in the included studies were categorised into feasibility, interrater reliability or validity. Table 3 presents the quality assessment for each psychometric property with summary scores ranging from 0.27 to 0.91 (summary scores also presented in Table 4). The study design was sufficiently described in six examinations, the method of selection in four examinations and the analytical methods in nine examinations.

**TABLE 3:** Quality assessment per psychometric property of the included studies.

References	Psychometric property^a^	1	2	3	4	5	6	7	8	9	10	11	12	13	14	Summary score
Bodfish et al. (1997)	Feasibility	No	Partial	Partial	Yes	NA	NA	NA	Yes	Yes	Yes	NA	Partial	Partial	Yes	0.70
Matson et al. (2002)	Feasibility	No	Partial	Partial	Yes	NA	NA	NA	Partial	Yes	Yes	NA	Yes	Partial	Yes	0.70
Ellis et al. (1996)	Interrater Reliability	Yes	Yes	Yes	Yes	NA	NA	NA	Yes	No	Yes	Yes	Partial	Yes	Yes	0.86
Bodfish et al. (1997)	Interrater Reliability	Yes	Partial	Partial	No	NA	NA	NA	Partial	Partial	Partial	Yes	No	Yes	Yes	0.59
Valdovinos et al. (2004)	Interrater Reliability	No	Partial	Partial	Yes	NA	NA	NA	Yes	No	Yes	Partial	No	Partial	No	0.45
Ricketts et al. (1995)	Interrater Reliability	No	Partial	Yes	Yes	NA	NA	NA	Partial	No	No	Partial	No	Partial	No	0.36
Newell et al. (2001)	Interrater Reliability	No	Partial	Partial	No	NA	NA	NA	No	Yes	Partial	No	Partial	Partial	No	0.32
Ganesh et al. (1989)	Interrater Reliability	No	Partial	Partial	Yes	NA	NA	NA	Partial	No	No	No	No	Partial	No	0.27
Garcia and Matson (2008)	Validity	Yes	Yes	Partial	Yes	NA	NA	NA	Yes	Yes	Yes	Yes	Yes	Yes	Yes	0.95
De Kuijper and Hoekstra (2016)	Validity	Yes	Yes	Partial	Yes	NA	NA	NA	Yes	Yes	Yes	Yes	Partial	Yes	Yes	0.91
Bodfish et al. (1997)	Validity	Yes	Yes	Partial	Yes	NA	NA	NA	Yes	Yes	Partial	Yes	Partial	Yes	Yes	0.86
Newell et al. (2001)	Validity	Partial	Yes	Yes	Yes	NA	NA	NA	Yes	Partial	Yes	Yes	Partial	Yes	Yes	0.86
Advokat et al. (2000)	Validity	Partial	Partial	Partial	Yes	NA	NA	NA	Yes	Partial	Yes	Yes	Yes	Yes	Yes	0.82
Ellis et al. (1996)	Validity	Yes	Yes	Yes	Yes	NA	NA	NA	Yes	No	Yes	No	Partial	Yes	Yes	0.77

*Note:* Items of the Standard Quality Assessment Criteria for Evaluating Primary Research Papers from a Variety of Fields (Kmet et al. 2004): 1. Question/objective sufficiently described? 2. Study design evident and appropriate? 3. Method of subject/comparison group selection or source of information/input variables described and appropriate? 4. Subject (and comparison group, if applicable) characteristics sufficiently described? 5. If interventional and random allocation was possible, was it described? 6. If interventional and blinding of investigators was possible, was it reported? 7. If interventional and blinding of subjects was possible, was it reported? 8. Outcome and (if applicable) exposure measure(s) well defined and robust to measurement/misclassification bias? Means of assessment reported? 9. Sample size appropriate? 10. Analytic methods described/justified and appropriate? 11. Some estimate of variance is reported for the main results? 12. Controlled for confounding? 13. Results reported in sufficient detail? 14. Conclusions supported by the results?

Abbreviation: NA, not applicable.

^a^ When multiple instruments were examined in one study we evaluated the quality per psychometric property rather than per instrument.

### 3.4 Data Synthesis of Psychometric Properties

The 10 included studies reported psychometric properties of seven different instruments to assess movement disorders in people with intellectual disabilities using antipsychotics. One instrument, the Matson Evaluation of Drug Side Effects (MEDS), had three subscales that assess movement disorders. An overview of the psychometric properties of these instruments and the summary score of the quality assessment is presented in Table 4 using the guidelines for interpretation as described in Table 1. Psychometric properties other than feasibility, interrater reliability or validity—for example, responsiveness or internal consistency—were not examined in any of the included studies. The instruments were employed for the assessment of different movement disorders. Instruments that solely assess dyskinesia were the Dyskinesia Identification System: Condensed User Scale (DISCUS); the AIMS; the Analogue functional analyses; and the CLAMPS Abnormal Movement Scale (CLAMPS). Instruments that only assess akathisia were the Akathisia Ratings of Movement Scale (ARMS), the Checklist for motor restlessness and the subscale Central Nervous System—Behavioural/Akathisia (CNS‐BA) of the MEDS. The subscale Central Nervous System—Dystonia (CNS‐D) of the MEDS was the only instrument to solely assess dystonia. There was no instrument that only assessed parkinsonism. However, one rating scale assessed a combination of parkinsonism and dyskinesia: the subscale Central Nervous System—Parkinsonism/Dyskinesia (CNS‐PD) of the MEDS. Descriptions of the seven instruments and the findings regarding their psychometric properties are presented in the sections below. More details on results of the individual studies, for example, which groups were compared, are presented in Table 2.

#### 3.4.1 DISCUS

The DISCUS is a 15‐item rating scale designed specifically for use in individuals with developmental disabilities. It combines ratings of frequency and severity of dyskinesia symptomatology in seven anatomical regions of the body. It contains nine oro‐bucco‐lingual items and six head‐trunk‐limb items (Sprague and Kalachnik 1991). Psychometric properties of the DISCUS were reported in five different studies. One study reported good (77%–100%) feasibility (Matson et al. 2002), whereas the other four studies did not report on feasibility. Interrater reliability was investigated in three studies and varied with ICC values negative (0.40) (Ellis et al. 1996) and positive (0.73–0.84) (Ellis et al. 1996; Newell et al. 2001; Ricketts et al. 1995). Validity was examined in three studies. Significant good correlations (0.56–0.69) were found with items from other rating scales assessing dyskinesia (AIMS and CLAMPS) (Ellis et al. 1996) and significant fair to good correlations within (0.394–0.641) and significant fair to strong correlations between (0.396–0.999) testing periods (Newell et al. 2001) were found. Furthermore, De Kuijper and Hoekstra (2016) compared the DISCUS with the AIMS and found that ratings of movement disorders based on assessment of intellectual disabilities physicians (medical doctors with a specialist postgraduate training program in the complex health needs, associated conditions and specific communication challenges faced by people with intellectual disabilities (Huisman et al. 2024)) were more in accordance with observations of caregivers for the DISCUS than for the AIMS (De Kuijper and Hoekstra 2016) (Table 4).

#### 3.4.2 AIMS

The AIMS is a global rating scale for tardive dyskinesia with ratings of presence and severity in seven different anatomical regions (Guy et al. 1976). The AIMS was examined in three different studies. Feasibility of the AIMS was not examined in the included studies. Interrater reliability was examined in two studies and they revealed conflicting results with values negative to positive (0.52–0.71) (Ellis et al. 1996) and positive (0.76) (Ricketts et al. 1995). To substantiate validity, in one study the AIMS was compared to other instruments that rate dyskinesia (CLAMPS and DISCUS); this resulted in good (0.52–0.56) Spearman correlation coefficients for total scores (Ellis et al. 1996). Another study comparing the AIMS with the DISCUS found that ratings of movement disorders based on assessment of intellectual disabilities physicians were less in accordance with observations of caregivers for the AIMS than the DISCUS (De Kuijper and Hoekstra 2016) (Table 4).

#### 3.4.3 Analogue Functional Analyses

Analogue functional analyses consisted of scoring occurrences of dyskinesia movements every 10 s from videotaped sessions using paper and pencil (Valdovinos et al. 2004). This instrument was examined in one study. Feasibility and validity were not examined in the included study. Results of interrater reliability analyses were positive (0.91–0.98) (Valdovinos et al. 2004) (Table 4).

#### 3.4.4 CLAMPS

The CLAMPS was specifically designed for the assessment of movement disorders in individuals with developmental disabilities. It consists of ratings for frequency and severity of tardive dyskinesia in seven anatomical regions of the body (Ellis et al. 1996). The CLAMPS was examined in one study. Feasibility was not examined. Interrater reliability for frequency ratings ranged from negative to positive (0.41–0.98) and for severity ratings ranged from negative to positive (0.22–0.71). To support validity, CLAMPS scores were compared to AIMS and DISCUS scores—other instruments that rate dyskinesia—which resulted in good (0.52–0.69) Spearman correlation coefficients for total scores (Ellis et al. 1996) (Table 4).

#### 3.4.5 ARMS

The ARMS is a 10‐item scale for the rating of akathisia and comprised of four sitting position items; three standing position items and three lying down position items (Bodfish et al. 1997). This instrument was examined in two studies. Feasibility was excellent (94%–100%) for items 1 to 7 (sitting and standing) and good (62%) for items 8 to 10 (lying down). Interrater reliability was negative (0.69) (Bodfish et al. 1997). Validity was supported by the ability of the ARMS to measure group differences (Bodfish et al. 1997), which was confirmed by another included study (Garcia and Matson 2008) (Table 4).

#### 3.4.6 Checklist for Motor Restlessness

For the Checklist for motor restlessness the authors used a slightly modified checklist of specific phenomena to record observable motor restlessness with 12 items in three categories (sitting, standing, lying) (Ganesh et al. 1989). It was examined in one study. Feasibility and validity were not examined. Interrater reliability was examined and positive (0.86–0.98) (Ganesh et al. 1989) (Table 4).

#### 3.4.7 MEDS—Subscale CNS‐BA

The MEDS is a 90‐item comprehensive measure of side effects of psychotropics. It is divided into nine subscales; one of those subscales is CNS‐BA. It consists of eight items which can be scored on severity and duration. It can be utilised to assess akathisia (Matson et al. 1998). This subscale of the MEDS was examined in three studies. Feasibility and interrater reliability of the MEDS CNS‐BA subscale were not investigated in the included studies. The ability of the CNS‐BA subscale to differentiate between groups (indicator of validity) was demonstrated by two studies (Advokat et al. 2000; Garcia and Matson 2008). Another study that compared ratings of intellectual disabilities physicians with observations by professional caregivers (indicated by results on the MEDS CNS‐BA subscale) showed that there was no complete agreement between intellectual disabilities physicians and professional caregivers (De Kuijper and Hoekstra 2016) (Table 4).

#### 3.4.8 MEDS—Subscale CNS‐D

Subscale CNS‐D of the MEDS consists of five items, which are scored on severity and duration (Matson et al. 1998). It can be utilised to assess dystonia. This subscale of the MEDS was investigated in one included study. Feasibility and interrater reliability of this subscale were not examined in the studies included in this systematic review. The included study revealed that CNS‐D was not able to significantly detect differences among groups (indicator of validity) (Advokat et al. 2000) (Table 4).

#### 3.4.9 MEDS—Subscale CNS‐PD

The subscale CNS‐PD of the MEDS combines the assessment of parkinsonism with the assessment of dyskinesia. There are 14 items in this subscale that are scored on severity and duration (Matson et al. 1998). This subscale was investigated in two included studies. Feasibility and interrater reliability of this subscale were not examined in the included studies. One included study indicated that the CNS‐PD was not able to significantly detect differences among groups (indicator of validity) (Advokat et al. 2000). And, similar to the CNS‐BS subscale of the MEDS, another included study revealed that there was no agreement between ratings of intellectual disabilities physicians with observations by professional caregivers (indicated by results on the MEDS CNS‐PD subscale) (De Kuijper and Hoekstra 2016) (Table 4).

**TABLE 4:** Data synthesis of psychometric properties of instruments to assess antipsychotic‐induced movement disorders.

Instrument	References	Psychometric properties^a^, ^b^			Summary score^c^
		Feasibility	Interrater Reliability	Validity	
DISCUS (dyskinesia)	De Kuijper and Hoekstra (2016)			No hypothesis DISCUS more reliable than AIMS.	0.91
	Ellis et al. (1996)		− to +	Hypothesis (subscales which measure tardive dyskinesia yield similar ratings) confirmed: SCC total DISCUS and total CLAMPS + SCC total DISCUS and total AIMS +	0.77–0.86
	Matson et al. (2002)	+			0.70
	Newell et al. (2001)		+	No hypothesis Significant interitem correlations within three testing periods; SCC +/− to + Significant interitem correlations between three testing periods; SCC +/− to ++ Significant predictor items were found.	0.32–0.86
	Ricketts et al. (1995)		+		0.36
AIMS (dyskinesia)	Ellis et al. (1996)		−	Hypothesis (subscales which measure tardive dyskinesia yield similar ratings) confirmed: SCC total AIMS and total CLAMPS + SCC total AIMS and total DISCUS +	0.77–0.86
	Ricketts et al. (1995)		+		0.36
	De Kuijper and Hoekstra (2016)			No hypothesis DISCUS more reliable than AIMS.	0.91
Analogue functional analyses (dyskinesia)	Valdovinos et al. (2004)		+		0.45
CLAMPS (dyskinesia)	Ellis et al. (1996)		Frequency: − to + Severity: − to +	Hypothesis (subscales which measure tardive dyskinesia yield similar ratings) confirmed: SCC total severity and total frequency CLAMPS ++ SCC total CLAMPS and total AIMS + SCC total CLAMPS and total DISCUS +	0.77–0.86
ARMS (akathisia)	Bodfish et al. (1997)	Items 1 to 7: ++ Items 8 to 10: +	−	Hypothesis (differences between certain groups as measured by ARMS) confirmed.	0.59–0.86
	Garcia and Matson (2008)			No hypothesis Significant differences on ARMS between groups. Two items differentiated between those with and without diagnosis. Pearson correlation between ARMS and MEDS CNS‐BA 0.51.	0.95
Checklist for motor restlessness (akathisia)	Ganesh et al. (1989)		+		0.27
MEDS subscale CNS‐BA (akathisia)	Advokat et al. (2000)			No hypothesis Ability to detect significant (and clinically relevant) differences on severity subscale CNS‐BA.	0.82
	De Kuijper and Hoekstra (2016)			No hypothesis Observations and ratings MEDS CNS‐BA do not completely agree with ratings ID physician.	0.91
	Garcia and Matson (2008)			No hypothesis Significant differences on MEDS CNS‐BA between groups. Four items of MEDS CNS‐BA differentiated between those with and without diagnosis. Pearson correlation between MEDS CNS‐BA and ARMS 0.51.	0.95
MEDS subscale CNS‐D (dystonia)	Advokat et al. (2000)			No hypothesis No significant differences for CNS‐D.	0.82
MEDS subscale CNS‐PD (parkinsonism and dyskinesia)	Advokat et al. (2000)			No hypothesis No significant differences for CNS‐PD.	0.82
	De Kuijper and Hoekstra (2016)			No hypothesis Observations and ratings MEDS CNS‐PD do not completely agree with ratings ID physician.	0.91

Abbreviations: AIMS, Abnormal Involuntary Movement Scale; ARMS, Akathisia Ratings of Movement Scale; CLAMPS; CLAMPS Abnormal Movement Scale; CNS‐BA, Central Nervous System‐Behavioural/Akathisia; CNS‐D, Central Nervous System‐Dystonia; CNS‐PD, Central Nervous System‐Parkinsonism/Dyskinesia; DISCUS, Dyskinesia Identification System Condensed User Scale; MEDS, Matson Evaluation of Drug Side Effects; SCC, Spearman correlation coefficient.

^a^ Psychometric properties as reported in the articles were converted as described in the Methods section.

^b^ Results of psychometric properties were summarised as described in the Methods section.

^c^ Summary score of the Standard Quality Assessment Criteria for Evaluating Primary Research Papers from a Variety of Fields (Kmet et al. 2004).

## 4 Discussion

To the best of our knowledge, this is the first systematic literature review to provide a comprehensive overview of the psychometric properties of instruments used to assess movement disorders in people with intellectual disabilities using antipsychotics. We identified ten studies that together evaluated the psychometric properties of seven instruments: DISCUS, AIMS, analogue functional analyses, CLAMPS, ARMS, checklist for motor restlessness and the MEDS. The MEDS had three subscales for the assessment of movement disorders (CNS‐BA, CNS‐D and CNS‐PD). Psychometric properties other than feasibility, interrater reliability or validity were not examined in any of the included studies. Feasibility was examined in two studies and was good for the DISCUS and good to excellent for the ARMS. Findings on interrater reliability and validity occasionally demonstrated varied results. Interrater reliability was determined for six out of seven instruments (DISCUS, AIMS, analogue functional analyses, CLAMPS, ARMS and the checklist for motor restlessness) and varied for the DISCUS, AIMS and CLAMPS. Validity was examined for five instruments (DISCUS, AIMS, CLAMPS, ARMS and the three subscales of the MEDS). Most studies showed evidence supporting their validity; however, for the MEDS subscales CNS‐BA, CNS‐D and CNS‐PD, there was no or conflicting evidence.

A previous scoping review examined instruments to measure side effects across all types of medication in people with intellectual disabilities (Kelly et al. 2022), not solely antipsychotics. They identified 14 instruments, of which only three were movement disorder‐related (ARMS, DISCUS and subscale CNS‐BA of the MEDS). These three instruments were also included in this systematic literature review. Variation in included studies between this scoping review and our systematic review can be attributed to methodological variation. For example, the scoping review included grey literature, whereas in our systematic review we did not. Additionally, we employed a broader search strategy in this systematic review compared to the scoping review, using extensive synonyms and alternative spellings. No restriction on publication date was applied in this systematic review, whereas the scoping review only included articles from the last 20 years. Comparison of the results revealed both similarities and discrepancies, attributable to our focus on antipsychotics rather than on all medications. Consistent with our results, the scoping review found that the ARMS was able to identify which participants had symptoms of akathisia. Additionally, we identified a study of the interrater reliability and feasibility of the ARMS (Bodfish et al. 1997), which was not covered in the scoping review. Contrary to our results, the scoping review reported no significant change in DISCUS scores following administration of antipsychotics. We identified studies reporting on validity with correlations ranging from moderate to strong (Ellis et al. 1996; Newell et al. 2001), indicating variable agreement of the DISCUS with other instruments assessing dyskinesia. Furthermore, additional evidence for feasibility and interrater reliability of the DISCUS was found in our systematic review. Finally, while the scoping review addressed the MEDS as a whole, only the CNS‐BA subscale had separate psychometric data. Consistent with our findings, the CNS‐BA subscale demonstrated the ability to identify which participants had symptoms of akathisia.

In this systematic literature review, we assessed the quality of the examination of psychometric properties in the included studies. Four studies only involved 2 to 7 participants (Ellis et al. 1996; Ganesh et al. 1989; Ricketts et al. 1995; Valdovinos et al. 2004). Such a small sample size significantly undermines the representativeness of the results. The study design was sufficiently described in six out of 14 examinations and the method of selection of participants only in four examinations. This diminishes the trustworthiness of the research and hinders reproducibility. These substantial limitations should be taken into account when interpreting the psychometric results.

### 4.1 Strengths and Limitations

Several strengths of this systematic review are noteworthy. First, we used a pre‐registered protocol and applied a transparent search strategy with clear documentation of the origin of the results presented, which enhances the reproducibility of the results. The comprehensive and structured search in multiple databases minimised the risk of missing relevant studies. To further minimise the risk of missing relevant studies, supplementary searches were conducted through review of the references of included studies and relevant systematic reviews. Second, strict inclusion criteria were applied: all participants were required to have an intellectual disability (IQ < 70) and all were required to use antipsychotics, with exceptions only for groups for the examination of validity. Since we solely included studies that examined psychometric properties, we had to exclude 43 studies that reported a total of 21 instruments to assess movement disorders in people with intellectual disabilities using antipsychotics that did not report on the psychometric properties of these instruments. Of the 21 instruments, only three (AIMS, DISCUS and MEDS subscales) were examined for their psychometric properties in the included studies in this systematic review. This illustrates that our strict inclusion criteria may have limited the number of eligible studies and instruments. However, it did ensure we only retrieved research regarding the psychometric properties of instruments used for assessing movement disorders in people with intellectual disabilities using antipsychotics, thereby enhancing the clinical relevance and specificity of the findings. Third, the screening of potential relevant studies was independently performed by two researchers, which reduced the chance of selection bias. And finally, we assessed the quality of the examination of the psychometric properties rather than the entire study, which provided insight into the transparency and trustworthiness of the research. A limitation of this systematic review is that conclusions are limited by the heterogeneity of the included studies. This substantial variability across studies reduced the strength of the evidence and made conducting a meta‐analysis unfeasible.

### 4.2 Clinical Implications and Future Research

This systematic review showed limited and varying evidence reflecting a gap in knowledge regarding the psychometric properties of instruments used to assess movement disorders in people with intellectual disabilities using antipsychotics. There is a great need for further high‐quality research to ensure the quality of the instruments and to provide evidence‐based recommendations for the selection of instruments with sufficient psychometric properties for clinical practice. This could lead to earlier and improved assessment and identification of antipsychotic‐induced movement disorders in people with intellectual disability. Adequate management of movement disorders may lead to less discomfort and improved quality of life. Eight out of 10 included studies were published more than two decades ago, which may reflect a decline of research interest and adds to the necessity of maintaining an up‐to‐date understanding of the psychometric properties of instruments used to assess antipsychotic‐induced movement disorders in people with intellectual disabilities. Notably, all identified instruments were based on caregiver or clinician observation. Observational assessment of psychomotor disturbances might be less sensitive and reliable than measurement instruments (Docx et al. 2015). Therefore, future research should also explore the development and validation of measurement instruments that clinicians could use to objectively measure movement disorders in people with intellectual disabilities using antipsychotics.

## 5 Conclusions

This systematic literature review included 10 studies that examined the psychometric properties of seven instruments used to assess movement disorders in people with intellectual disabilities using antipsychotics. The identified instruments were: DISCUS, AIMS, analogue functional analyses, CLAMPS, ARMS, checklist for motor restlessness and MEDS subscales CNS‐BA, CNS‐D and CND‐PD. For these instruments, only feasibility, interrater reliability and validity were examined. There was not a singular instrument that consistently demonstrated good feasibility, interrater reliability and validity across studies. Considering the importance of early side effect monitoring and subsequent proper management, future research should therefore prioritise the evaluation of existing instruments and the systematic examination of their psychometric properties in people with intellectual disabilities. Moreover, future research is warranted for the development and validation of novel objective instruments measuring movement disorders in people with intellectual disabilities using antipsychotics.

## Author Contributions

Conception and design: Maria Louise Dieudonnée Hoekstra‐van Duijn, Sylvie Beumer and Dederieke Anne Maria Maes‐Festen. Acquisition of data: Maria Louise Dieudonnée Hoekstra‐van Duijn, Maureen Bernardina Gerhardus Wissing and Bibi den Haan. Analysis and interpretation of data: Maria Louise Dieudonnée Hoekstra‐van Duijn, Maureen Bernardina Gerhardus Wissing, Bibi den Haan, Sylvie Beumer and Dederieke Anne Maria Maes‐Festen. Drafting the manuscript: Maria Louise Dieudonnée Hoekstra‐van Duijn and Maureen Bernardina Gerhardus Wissing. Revising the manuscript critically: Maria Louise Dieudonnée Hoekstra‐van Duijn, Maureen Bernardina Gerhardus Wissing, Bibi den Haan, Sylvie Beumer and Dederieke Anne Maria Maes‐Festen.

## Funding

This work was supported by the Academic Collaborative Research Center Healthy Ageing and Intellectual Disabilities (HA‐ID). The Academic Collaborative Research Center HA‐ID is financially supported by three Dutch residential care facilities (Abrona, Amarant, and Ipse de Bruggen), and the Department of General Practice, Intellectual Disability Medicine of the Erasmus MC—University Medical Center Rotterdam. The funding source was not involved in the conduct of the research and/or preparation of the manuscript and is not liable for any use that may be made of the information contained therein.

## Conflicts of Interest

The authors declare no conflicts of interest.

## Supporting information


**Data S1: Supplement A.** Search strategy electronic databases.
**Supplement B.** Data extraction form.

## Data Availability

The data that support the findings of this study are available from the corresponding author upon reasonable request.
