# Supplementary material for: Psychometric Properties of Instruments to Assess Movement Disorders in People With Intellectual Disabilities Using Antipsychotics: A Systematic Literature Review
Source: J Appl Res Intellect Disabil. 2026 Sep 8;39(5):e70303. doi: 10.1111/jar.70303 (PMC13552245; doi:10.1111/jar.70303)
Supplement: Supplementary file 1 [file JAR-39-e70303-s001.docx]

**Supplementary material**

**Supplement A – Search strategy electronic databases**

**embase.com**

('intellectual impairment'/de OR 'mental deficiency'/de OR 'learning disorder'/de OR 'developmental disorder'/de OR 'mentally disabled person'/de OR (((intel* OR mental* OR learning) NEAR/3 (impair* OR deficien* OR retard* OR disab* OR disorder* OR handicap* OR deficit* OR defect*)) OR ((cognit* OR developmental*) NEAR/3 (deficien* OR retard* OR disab* OR handicap* OR deficit* OR challenged OR defect*)) OR idiocy):ab,ti) AND ('dystonia'/exp OR 'dystonic disorder'/de OR 'akathisia'/de OR 'parkinsonism'/de OR 'extrapyramidal syndrome'/de OR 'extrapyramidal symptom'/de OR 'dyskinesia'/exp OR ('motor dysfunction'/de AND ('drug induced disease'/de OR 'adverse drug reaction'/de OR 'neuroleptic agent'/mj)) OR (dystoni* OR acathisi* OR restless* OR akathisia* OR parkinsonism* OR parkinsonian* OR extrapyramid* OR extra-pyramid* OR dyskin* OR ((motor OR psychomotor OR movement*) NEAR/6 (dysfunction* OR disorder* OR symptom* OR side-effect* OR agitat*) AND (drug-induc* OR antipsycho* OR anti-psycho* OR neuroleptic*))):ab,ti) AND ('validation study'/de OR 'comparative study'/de OR 'psychometry'/exp OR 'outcome assessment'/exp OR 'observer variation'/exp OR 'interrater reliability'/de OR 'intrarater reliability'/de OR 'health status indicator'/de OR 'reproducibility'/exp OR 'discriminant analysis'/exp OR 'coefficient of variation'/de OR 'internal consistency'/de OR 'instrument validation'/de *OR 'rating scale'/de OR 'scoring system'/de* OR 'devices'/de OR (instrument* OR psychometr* OR clinimetr* OR clinometr* OR (outcome NEAR/3 (assessment* OR measure*)) OR (observer* NEAR/3 variation*) OR reproducib* OR reliab* OR unreliab* OR valid* OR coefficient OR homogeneity OR homogeneous OR (internal* NEAR/3 consisten*) OR (cronbach* NEAR/3 (alpha OR alphas)) OR (item NEAR/3 (correlation* OR selection* OR reduction*)) OR agreement OR precision OR imprecision OR (precise* NEAR/3 value*) OR (test NEAR/3 retest) OR (reliab* NEAR/3 (test OR retest)) OR stabilit* OR interrater* OR inter-rater* OR intrarater* OR intra-rater* OR intertester* OR inter-tester* OR intratester* OR intra-tester* OR interobserver* OR inter-observer* OR intraobserver* OR intra-observer* OR intertechnician* OR inter-technician* OR intratechnician* OR intra-technician* OR interexaminer* OR inter-examiner* OR intraexaminer* OR intra-examiner* OR interassay* OR inter-assay* OR intraassay* OR intra-assay* OR interindividual* OR inter-individual* OR intraindividual* OR intra-individual* OR interparticipant* OR inter-participant* OR intraparticipant* OR intra-participant* OR kappa OR kappa-s OR kappas OR repeatab* OR ((replicab* OR repeated) NEAR/3 (measure OR measures OR findings OR result OR results OR test OR tests)) OR generaliza* OR generalisa* OR concordance OR (intraclass NEAR/3 correlation*) OR discriminative OR 'known group' OR (factor NEXT/1 (analys* OR structure*)) OR dimension* OR subscale* OR (multitrait NEAR/3 scaling NEAR/3 (analysis OR analyses)) OR 'item discriminant' OR (interscale NEAR/3 correlation*) *OR ((error OR errors) NOT (inborn-error*))* OR (((individual OR interval OR rate OR analysis OR values) NEAR/3 variabil*) NOT heart-rate*) OR (uncertainty NEAR/3 (measurement OR measuring)) OR 'standard error of measurement' OR sensitiv* OR *responsiveness** OR (limit NEAR/3 detection) OR 'minimal detectable concentration' OR interpretab* OR ((minimal OR minimally OR clinical OR clinically) NEAR/3 (important OR significant OR detectable) NEAR/3 (change OR difference)) OR (small* NEAR/3 (real OR detectable) NEAR/3 (change OR difference)) OR 'meaningful change' OR 'ceiling effect' OR 'floor effect' OR 'Item response model' OR IRT OR Rasch OR 'Differential item functioning' OR DIF OR 'computer adaptive testing' OR 'item bank' OR 'cross-cultural equivalence' *OR rating* OR scale**):ab,ti) NOT ([animals]/lim NOT [humans]/lim) NOT ([Conference Abstract]/lim) AND [English]/lim

**Medline Ovid**

(Intellectual Disability/ OR mental deficiency/ OR Learning Disorders/ OR Developmental Disabilities/ OR Mentally Disabled Persons/ OR (((intel* OR mental* OR learning) ADJ3 (impair* OR deficien* OR retard* OR disab* OR disorder* OR handicap* OR deficit* OR defect*)) OR ((cognit* OR developmental*) ADJ3 (deficien* OR retard* OR disab* OR handicap* OR deficit* OR challenged OR defect*)) OR idiocy).ab,ti.) AND (Dystonia/ OR Dystonic Disorders/ OR exp Psychomotor Agitation/ OR Parkinsonian Disorders/ OR exp Dyskinesias/ OR ((Movement Disorders/ OR Psychomotor Disorders/) AND ("Drug-Related Side Effects and Adverse Reactions"/ OR * Antipsychotic Agents/)) OR (dystoni* OR acathisi* OR restless* OR akathisia* OR parkinsonism* OR parkinsonian* OR extrapyramid* OR extra-pyramid* OR dyskin* OR ((motor OR psychomotor OR movement*) ADJ6 (dysfunction* OR disorder* OR symptom* OR side-effect* OR agitat*) AND (drug-induc* OR antipsycho* OR anti-psycho* OR neuroleptic*))).ab,ti.) AND (instrumentation.xs. OR methods.xs. OR exp "Validation Studies"/ OR exp "Comparative Study"/ OR exp "psychometrics"/ OR "outcome assessment (health care)"/ OR exp "observer variation"/ OR exp "Health Status Indicators"/ OR exp "reproducibility of results"/ OR exp "discriminant analysis"/ OR (instrument* OR psychometr* OR clinimetr* OR clinometr* OR (outcome ADJ3 (assessment* OR measure*)) OR (observer* ADJ3 variation*) OR reproducib* OR reliab* OR unreliab* OR valid* OR coefficient OR homogeneity OR homogeneous OR (internal* ADJ3 consisten*) OR (cronbach* ADJ3 (alpha OR alphas)) OR (item ADJ3 (correlation* OR selection* OR reduction*)) OR agreement OR precision OR imprecision OR (precise* ADJ3 value*) OR (test ADJ3 retest) OR (reliab* ADJ3 (test OR retest)) OR stabilit* OR interrater* OR inter-rater* OR intrarater* OR intra-rater* OR intertester* OR inter-tester* OR intratester* OR intra-tester* OR interobserver* OR inter-observer* OR intraobserver* OR intra-observer* OR intertechnician* OR inter-technician* OR intratechnician* OR intra-technician* OR interexaminer* OR inter-examiner* OR intraexaminer* OR intra-examiner* OR interassay* OR inter-assay* OR intraassay* OR intra-assay* OR interindividual* OR inter-individual* OR intraindividual* OR intra-individual* OR interparticipant* OR inter-participant* OR intraparticipant* OR intra-participant* OR kappa OR kappa-s OR kappas OR repeatab* OR ((replicab* OR repeated) ADJ3 (measure OR measures OR findings OR result OR results OR test OR tests)) OR generaliza* OR generalisa* OR concordance OR (intraclass ADJ3 correlation*) OR discriminative OR known group OR (factor ADJ (analys* OR structure*)) OR dimension* OR subscale* OR (multitrait ADJ3 scaling ADJ3 (analysis OR analyses)) OR item discriminant OR (interscale ADJ3 correlation*) OR ((error OR errors) NOT (inborn-error*)) OR (((individual OR interval OR rate OR analysis OR values) ADJ3 variabil*) NOT heart-rate*) OR (uncertainty ADJ3 (measurement OR measuring)) OR standard error of measurement OR sensitiv* OR responsiveness* OR (limit ADJ3 detection) OR minimal detectable concentration OR interpretab* OR ((minimal OR minimally OR clinical OR clinically) ADJ3 (important OR significant OR detectable) ADJ3 (change OR difference)) OR (small* ADJ3 (real OR detectable) ADJ3 (change OR difference)) OR meaningful change OR ceiling effect OR floor effect OR Item response model OR IRT OR Rasch OR Differential item functioning OR DIF OR computer adaptive testing OR item bank OR cross-cultural equivalence OR rating* OR scale*).ab,ti.) NOT (exp animals/ NOT humans/) AND english.la.

**Web of science**

TS=(((((intel* OR mental* OR learning) NEAR/2 (impair* OR deficien* OR retard* OR disab* OR disorder* OR handicap* OR deficit* OR defect*)) OR ((cognit* OR developmental*) NEAR/2 (deficien* OR retard* OR disab* OR handicap* OR deficit* OR challenged OR defect*)) OR idiocy)) AND ((dystoni* OR acathisi* OR restless* OR akathisia* OR parkinsonism* OR parkinsonian* OR extrapyramid* OR extra-pyramid* OR dyskin* OR ((motor OR psychomotor OR movement*) NEAR/5 (dysfunction* OR disorder* OR symptom* OR side-effect* OR agitat*) AND (drug-induc* OR antipsycho* OR anti-psycho* OR neuroleptic*)))) AND ((instrument* OR psychometr* OR clinimetr* OR clinometr* OR (outcome NEAR/2 (assessment* OR measure*)) OR (observer* NEAR/2 variation*) OR reproducib* OR reliab* OR unreliab* OR valid* OR coefficient OR homogeneity OR homogeneous OR (internal* NEAR/2 consisten*) OR (cronbach* NEAR/2 (alpha OR alphas)) OR (item NEAR/2 (correlation* OR selection* OR reduction*)) OR agreement OR precision OR imprecision OR (precise* NEAR/2 value*) OR (test NEAR/2 retest) OR (reliab* NEAR/2 (test OR retest)) OR stabilit* OR interrater* OR inter-rater* OR intrarater* OR intra-rater* OR intertester* OR inter-tester* OR intratester* OR intra-tester* OR interobserver* OR inter-observer* OR intraobserver* OR intra-observer* OR intertechnician* OR inter-technician* OR intratechnician* OR intra-technician* OR interexaminer* OR inter-examiner* OR intraexaminer* OR intra-examiner* OR interassay* OR inter-assay* OR intraassay* OR intra-assay* OR interindividual* OR inter-individual* OR intraindividual* OR intra-individual* OR interparticipant* OR inter-participant* OR intraparticipant* OR intra-participant* OR kappa OR kappa-s OR kappas OR repeatab* OR ((replicab* OR repeated) NEAR/2 (measure OR measures OR findings OR result OR results OR test OR tests)) OR generaliza* OR generalisa* OR concordance OR (intraclass NEAR/2 correlation*) OR discriminative OR "known group" OR (factor NEAR/1 (analys* OR structure*)) OR dimension* OR subscale* OR (multitrait NEAR/2 scaling NEAR/2 (analysis OR analyses)) OR "item discriminant" OR (interscale NEAR/2 correlation*) OR ((error OR errors) NOT (inborn-error*)) OR (((individual OR interval OR rate OR analysis OR values) NEAR/2 variabil*) NOT heart-rate*) OR (uncertainty NEAR/2 (measurement OR measuring)) OR "standard error of measurement" OR sensitiv* OR responsiveness* OR (limit NEAR/2 detection) OR "minimal detectable concentration" OR interpretab* OR ((minimal OR minimally OR clinical OR clinically) NEAR/2 (important OR significant OR detectable) NEAR/2 (change OR difference)) OR (small* NEAR/2 (real OR detectable) NEAR/2 (change OR difference)) OR "meaningful change" OR "ceiling effect" OR "floor effect" OR "Item response model" OR IRT OR Rasch OR "Differential item functioning" OR DIF OR "computer adaptivetesting" OR "item bank" OR "cross-cultural equivalence" OR rating* OR scale*))) AND DT=(article) AND LA=(english)

**Cochrane CENTRAL**

((((intel* OR mental* OR learning) NEAR/3 (impair* OR deficien* OR retard* OR disab* OR disorder* OR handicap* OR deficit* OR defect*)) OR ((cognit* OR developmental*) NEAR/3 (deficien* OR retard* OR disab* OR handicap* OR deficit* OR challenged OR defect*)) OR idiocy):ab,ti) AND ((dystoni* OR acathisi* OR restless* OR akathisia* OR parkinsonism* OR parkinsonian* OR extrapyramid* OR extra-pyramid* OR dyskin* OR ((motor OR psychomotor OR movement*) NEAR/6 (dysfunction* OR disorder* OR symptom* OR side-effect* OR agitat*) AND (drug-induc* OR antipsycho* OR anti-psycho* OR neuroleptic*))):ab,ti) AND ((instrument* OR psychometr* OR clinimetr* OR clinometr* OR (outcome NEAR/3 (assessment* OR measure*)) OR (observer* NEAR/3 variation*) OR reproducib* OR reliab* OR unreliab* OR valid* OR coefficient OR homogeneity OR homogeneous OR (internal* NEAR/3 consisten*) OR (cronbach* NEAR/3 (alpha OR alphas)) OR (item NEAR/3 (correlation* OR selection* OR reduction*)) OR agreement OR precision OR imprecision OR (precise* NEAR/3 value*) OR (test NEAR/3 retest) OR (reliab* NEAR/3 (test OR retest)) OR stabilit* OR interrater* OR inter-rater* OR intrarater* OR intra-rater* OR intertester* OR inter-tester* OR intratester* OR intra-tester* OR interobserver* OR inter-observer* OR intraobserver* OR intra-observer* OR intertechnician* OR inter-technician* OR intratechnician* OR intra-technician* OR interexaminer* OR inter-examiner* OR intraexaminer* OR intra-examiner* OR interassay* OR inter-assay* OR intraassay* OR intra-assay* OR interindividual* OR inter-individual* OR intraindividual* OR intra-individual* OR interparticipant* OR inter-participant* OR intraparticipant* OR intra-participant* OR kappa OR kappa-s OR kappas OR repeatab* OR ((replicab* OR repeated) NEAR/3 (measure OR measures OR findings OR result OR results OR test OR tests)) OR generaliza* OR generalisa* OR concordance OR (intraclass NEAR/3 correlation*) OR discriminative OR 'known group' OR (factor NEXT/1 (analys* OR structure*)) OR dimension* OR subscale* OR (multitrait NEAR/3 scaling NEAR/3 (analysis OR analyses)) OR 'item discriminant' OR (interscale NEAR/3 correlation*) *OR ((error OR errors) NOT (inborn-error*))* OR (((individual OR interval OR rate OR analysis OR values) NEAR/3 variabil*) NOT heart-rate*) OR (uncertainty NEAR/3 (measurement OR measuring)) OR 'standard error of measurement' OR sensitiv* OR *responsiveness** OR (limit NEAR/3 detection) OR 'minimal detectable concentration' OR interpretab* OR ((minimal OR minimally OR clinical OR clinically) NEAR/3 (important OR significant OR detectable) NEAR/3 (change OR difference)) OR (small* NEAR/3 (real OR detectable) NEAR/3 (change OR difference)) OR 'meaningful change' OR 'ceiling effect' OR 'floor effect' OR 'Item response model' OR IRT OR Rasch OR 'Differential item functioning' OR DIF OR 'computer adaptive testing' OR 'item bank' OR 'cross-cultural equivalence' *OR rating* OR scale**):ab,ti)

**PsycINFO Ovid**

(exp Intellectual Development Disorder/ OR Learning Disorders/ OR Developmental Disabilities/ OR (((intel* OR mental* OR learning) ADJ3 (impair* OR deficien* OR retard* OR disab* OR disorder* OR handicap* OR deficit* OR defect*)) OR ((cognit* OR developmental*) ADJ3 (deficien* OR retard* OR disab* OR handicap* OR deficit* OR challenged OR defect*)) OR idiocy).ab,ti.) AND (Muscular Disorders/ OR exp Dyskinesia/ OR ((Movement Disorders/ ) AND ("Side Effects (Drug)"/ OR Neuroleptic Drugs/)) OR (dystoni* OR acathisi* OR restless* OR akathisia* OR parkinsonism* OR parkinsonian* OR extrapyramid* OR extra-pyramid* OR dyskin* OR ((motor OR psychomotor OR movement*) ADJ6 (dysfunction* OR disorder* OR symptom* OR side-effect* OR agitat*) AND (drug-induc* OR antipsycho* OR anti-psycho* OR neuroleptic*))).ab,ti.) AND (exp "psychometrics"/ OR exp " Interrater Reliability"/ OR exp "Test Reliability"/ OR (instrument* OR psychometr* OR clinimetr* OR clinometr* OR (outcome ADJ3 (assessment* OR measure*)) OR (observer* ADJ3 variation*) OR reproducib* OR reliab* OR unreliab* OR valid* OR coefficient OR homogeneity OR homogeneous OR (internal* ADJ3 consisten*) OR (cronbach* ADJ3 (alpha OR alphas)) OR (item ADJ3 (correlation* OR selection* OR reduction*)) OR agreement OR precision OR imprecision OR (precise* ADJ3 value*) OR (test ADJ3 retest) OR (reliab* ADJ3 (test OR retest)) OR stabilit* OR interrater* OR inter-rater* OR intrarater* OR intra-rater* OR intertester* OR inter-tester* OR intratester* OR intra-tester* OR interobserver* OR inter-observer* OR intraobserver* OR intra-observer* OR intertechnician* OR inter-technician* OR intratechnician* OR intra-technician* OR interexaminer* OR inter-examiner* OR intraexaminer* OR intra-examiner* OR interassay* OR inter-assay* OR intraassay* OR intra-assay* OR interindividual* OR inter-individual* OR intraindividual* OR intra-individual* OR interparticipant* OR inter-participant* OR intraparticipant* OR intra-participant* OR kappa OR kappa-s OR kappas OR repeatab* OR ((replicab* OR repeated) ADJ3 (measure OR measures OR findings OR result OR results OR test OR tests)) OR generaliza* OR generalisa* OR concordance OR (intraclass ADJ3 correlation*) OR discriminative OR known group OR (factor ADJ (analys* OR structure*)) OR dimension* OR subscale* OR (multitrait ADJ3 scaling ADJ3 (analysis OR analyses)) OR item discriminant OR (interscale ADJ3 correlation*) OR ((error OR errors) NOT (inborn-error*)) OR (((individual OR interval OR rate OR analysis OR values) ADJ3 variabil*) NOT heart-rate*) OR (uncertainty ADJ3 (measurement OR measuring)) OR standard error of measurement OR sensitiv* OR responsiveness* OR (limit ADJ3 detection) OR minimal detectable concentration OR interpretab* OR ((minimal OR minimally OR clinical OR clinically) ADJ3 (important OR significant OR detectable) ADJ3 (change OR difference)) OR (small* ADJ3 (real OR detectable) ADJ3 (change OR difference)) OR meaningful change OR ceiling effect OR floor effect OR Item response model OR IRT OR Rasch OR Differential item functioning OR DIF OR computer adaptive testing OR item bank OR cross-cultural equivalence OR rating* OR scale*).ab,ti.) NOT (news OR congres* OR abstract* OR book* OR chapter* OR dissertation abstract*).pt. NOT (exp animals/ NOT humans/) AND english.la.

**Supplement B – Data extraction form**

| **GENERAL CHARACTERISTICS** |  |
| --- | --- |
| Author |  |
| Year of publication |  |
| Primary study objective(s) |  |
| Sample size |  |
| **PARTICIPANT CHARACTERISTICS** |  |
| Age | Mean age (SD; range) of participants with ID |
| Sex | N and/or % of female participants with ID |
| Antipsychotic use |  |
| Level of ID | N and/or %  Categories: mild (IQ 55-70); moderate (IQ 35-55); severe (IQ 25-35); profound (IQ <25); unspecified |
| Aetiology of ID |  |
| **INSTRUMENT CHARACTERISTICS** |  |
| Name of instrument_1 |  |
| Description of instrument_1 |  |
| Psychometric property of instrument_1 |  |
| Description of psychometric property_1 |  |
| Results of psychometric property_1 |  |
| [and so on for instrument 2, 3, 4, 5, and 6] | |
